# Rationale, design and methods for the 22 year follow-up of the Western Australian Pregnancy Cohort (Raine) Study

**DOI:** 10.1186/s12889-015-1944-6

**Published:** 2015-07-14

**Authors:** Leon M. Straker, Graham L. Hall, Jenny Mountain, Erin K. Howie, Elisha White, Nigel McArdle, Peter R. Eastwood, the Raine Study 22 year follow-up Investigator Group

**Affiliations:** 1 grid.1032.00000000403754078School of Physiotherapy and Exercise Science, Curtin University, GPO Box U1987, Perth, WA 6845 Australia; 2 grid.414659.b0000000088281230Telethon Kids Institute, University of Western Australia, Crawley, Australia; 3 grid.1012.20000000419367910The University of Western Australia, Perth, WA Australia; 4 grid.1032.00000000403754078Curtin University, Perth, WA Australia; 5 grid.416195.e0000000404533875Royal Perth Hospital, Perth, WA Australia

**Keywords:** Longitudinal cohort, Life-course, Spinal pain, Asthma, Sleep, Sleep disorders, Activity, Work absenteeism, Work presenteeism, Chronic disease, Raine Study

## Abstract

**Background:**

Young adulthood is a critical life period for health and health behaviours. Related measurements collected before and after birth, and during childhood and adolescence can provide a life-course analysis of important factors that contribute to health and behaviour in young adulthood. The Western Australian Pregnancy Cohort (Raine) Study has collected a large number of such measurements during the fetal, perinatal, infancy, childhood and adolescence periods and plans to relate them to common health issues and behaviours in young adults, including spinal pain, asthma, sleep disorders, physical activity and sedentary behaviour and, work absenteeism and presenteeism. The aim of this paper is to describe the rationale, design and methods of the 22 year follow-up of the Raine Study cohort.

**Methods/Design:**

The Raine Study is a prospective cohort study. Participants still active in the cohort (*n* = 2,086) were contacted around the time of their 22nd birthday and invited to participate in the 22 year follow-up. Each was asked to complete a questionnaire, attend a research facility for physical assessment and an overnight sleep study, wear activity monitors for a week, and to maintain a sleep and activity diary over this week. The questionnaire was broad and included questions related to sociodemographics, medical history, quality of life, psychological factors, lifestyle factors, spinal pain, respiratory, sleep, activity and work factors. Physical assessments included anthropometry, blood pressure, back muscle endurance, tissue sensitivity, lung function, airway reactivity, allergic status, 3D facial photographs, cognitive function, and overnight polysomnography.

**Discussion:**

Describing the prevalence of these health issues and behaviours in young adulthood will enable better recognition of the issues and planning of health care resources. Providing a detailed description of the phenotype of these issues will provide valuable information to help educate health professionals of the needs of young adults. Understanding the life-course risk factors of health issues and behaviours in young adulthood will have important health planning implications, supporting the development of targeted interventions to improve current health status and reduce the onset and development of further ill-health across adulthood.

## Background

### The Raine study

The Western Australian Pregnancy Cohort (Raine) Study is one of the largest, successful, prospective cohorts of pregnancy, childhood and adolescence to be carried out anywhere in the world. The participants, born between May 1989 and November 1991, have been assessed twelve times since their mothers were recruited at 16 to 18 weeks of pregnancy. As young adults, over 70 % of the participants are still actively engaged in the study.

The Raine Study started as a randomised controlled trial to examine the effects of frequent and repeated ultrasound scans on pregnancy outcomes [1]. Pregnant women were recruited from the public antenatal clinic at King Edward Memorial Hospital (the sole tertiary women’s and infant’s hospital in Perth, Western Australia) and nearby private practice clinics. Only those booked into clinics before 18 weeks gestation were included, in order to reduce bias from those referred in late pregnancy due to complications [2]. The study collected questionnaire information at 18 and 34 weeks’ gestation, in addition to ultrasound and Doppler information, mother and father information and pregnancy outcomes.

The Raine Study cohort was recognised as having great potential to inform developmental origins of health and disease [2] and, therefore, initial consent sought approval for long term follow-up. From the 2,900 women enrolled into the study there were 2,868 live births from 2826 mothers. The children have had data collected at 1, 2, 3, 5, 14, 17, 18, 20 and now 22 years of age. Available data cover a wide range of health and behaviour variables, reflecting the diverse research issues previously investigated. Currently, over 85,000 phenotypic, behavioural and environmental variables are available for each participant, along with an extensive genetics database. Biological samples were obtained from participants at many of these time points. The Raine cohort is well established and there is frequent contact between enrolled families and study organizers. A representative group of cohort members participates in ongoing discussions about research in the cohort.

### Young adulthood a critical period for health and related behaviours

The focus of the Raine Study has evolved from a ‘developmental origins’ approach to a ‘life-course’ approach, to allow investigation of the health and behaviours of the cohort in young adulthood and beyond, and to examine both accumulated risk and critical risk periods [3, 4]. Young adulthood is recognised as a critical life phase for health [5], and for physical and mental development [5, 6]. Young adulthood is a transitional time when health behaviours impact on adult lifestyle habits [5]. Young adults are at greater risk for health impacts and health outcomes than adolescents [7].

With Raine Study participants turning 22–23 years of age, five focus areas received funding for further, in-depth assessment: spinal pain, asthma, sleep and its disorders, physical activity and sedentary behaviour, and work productivity. These are important health-related issues, currently under-researched among young adults, which are likely to be influenced by early life factors and thus be informed by a life-course approach where rich pregnancy, perinatal, infancy, childhood and adolescent data are available.

### Spinal pain

#### The importance of spinal pain

Back pain and neck pain are, respectively, the 1st and 4th most common reasons for years lived with a disability [8]. One month prevalence for either back or neck pain has been estimated at 13–50 % in adults [9]. For the individual with disabling spinal pain the burden is substantial, including the experience of pain, restricted work/leisure activity, reduced quality of life, impaired general health and associated comorbidities such as anxiety and depression [9]. Back pain and neck pain both result in substantial financial costs for society through direct healthcare costs plus lost productivity. For example, back pain is one of the most expensive health care problems internationally [9]: in Australia for 2001, estimated costs were over $474 per capita [10].

#### Early life predictors of spinal pain in young adults

Contrary to popular belief, adolescents can experience disabling spinal pain [11]. While there is some limited evidence that the experience of spinal pain during adolescence increases the risk of spinal pain in adulthood [12], there is also a belief that adolescent spinal pain is transitory and part of normal development [13]. There is growing evidence to suggest that shared early life factors increase the risk of developing pain in later life [14, 15] including genetic and environmental factors. Life stress events, poor family functioning, low socioeconomic status, sleep disturbances, depression and anxiety potentially alter the function of the HPA axis leading to altered pain processing and increased stress sensitivity [16]. Factors such as high levels of adiposity, altered body postures and muscle deficits may interact with activity patterns, potentially, to promote repetitive strain on pain sensitive structures [14, 17]. These biopsychosocial factors develop in early life and may set risk trajectories for disabling spinal pain in adulthood [17]. Whilst several, longitudinal studies have identified biopsychosocial factors in adolescence which predict adult spinal pain [12, 15], the majority of studies has been uni-dimensional. To date, there are no pregnancy and birth cohort studies investigating vulnerability to disabling spinal pain in adulthood across the entire life-course.

#### Limited information about spinal pain disorders in young adults

Disability relating to spinal pain has been reported to be the most common cause of activity limitation below the age of 45 [18]. Despite this, there is very limited information about disabling spinal pain in young adults. A recent study reported that low back pain disability levels were constant between the ages of 20–40 years, although the 20–29 year age group were under represented in the study [19]. Disabling low back pain has also recently been identified in a small cohort study of young female nurses (mean age 22, *n* = 199) suggesting the behaviours associated with disabling low back pain may already be developed in adults in their early 20s [20]. Currently, there is no investigation of disabling spinal pain in young adults who are representative of the broader community. Additionally, it is not clear whether disabling spinal pain in adolescence tracks through to adulthood. This knowledge gap is critical [17] as it is not known whether adolescent and adult disabling spinal pain disorders are separate entities or part of an emerging disabling spinal pain vulnerability that develops in early life.

Despite the enormous cost, the prevention and management of disabling low back pain remains largely ineffective [21], with studies examining prevention interventions typically finding no positive effect [21]. Leading researchers have called for a cessation of all RCTs into managing spinal pain until there is a better understanding of key aspects of spinal pain including the life-course development, multi-dimensional characterisation and potential heterogeneity of the disorder [22, 23]. A well-characterised, longitudinal cohort, such as that in the Raine Study, is required to enable investigation into the multi-dimensional life-course risk factors and development of disabling spinal pain, and adequate capture of the multi-dimensional characteristics and potential heterogeneity of disabling spinal pain.

### Asthma

#### The importance of asthma disorders

Asthma is a chronic airways disease characterised by airway inflammation, recurrent respiratory symptoms, airway reactivity and airway obstruction that is at least partially reversible. According to the World Health Organisation, asthma affects over 235 million people and results in over 200,000 deaths each year, worldwide [24, 25]. Asthma is a heterogeneous disease and includes multiple phenotypes, with differing underlying disease pathogeneses, commonly recognised phenotypes include allergic asthma, non-allergic asthma, late-onset asthma, and asthma with obesity [26].

#### Early life risk factors for asthma

It is increasingly evident that lung function tracks into adult life [27], suggesting that environmental exposures (including maternal nutrition, environmental tobacco smoke, respiratory infections and allergen exposure) [28] in early life play a significant role in the development of chronic respiratory disease in adults. There is also emerging evidence that chronic obstructive pulmonary disease is linked to asthma in childhood [7, 29], further highlighting the importance and necessity of a true life course approach to the investigation of chronic respiratory diseases, such as asthma. Examples of the value of a life-course approach to understanding asthma can be seen in the following findings from the Raine and other cohort studies: (i) maternal nutrition and micro-nutrition and slower fetal growth is associated with lower lung function and increased atopy (predisposition to allergy), wheeze and asthma in childhood, [30, 31] and increased airway reactivity in adolescence [32]; (ii) wheezing and lower respiratory illness (LRI) in early life and atopy are independently associated with current asthma at 6 years [33]; (iii) recent wheeze and airway reactivity during childhood are independently associated with reduced lung growth during adolescence [34]; (iv) young adults with asthma have lower lung function than their healthy counterparts at age 19 and there is an accelerated decline in lung function in these individuals [35]; (v) prenatal growth is one potential risk factor linked to immunological development, which is related to long-term trends in airway reactivity and lung function [27, 36].

The persistence of asthma through childhood and into adult life is common in atopic individuals with low lung function and airway reactivity [37], however, these relationships are not always evident which suggests as yet unknown modifying pathways may be involved. Data from the Raine Study at the 14 year follow up reported that ~50 % of the atopic asthmatics did not demonstrate airway reactivity [38]. Furthermore, atopic asthmatics without airway reactivity had significantly lower total and house dust mite specific IgE levels compared with atopic asthmatics with airway reactivity. These data suggest a threshold level of specific IgE beyond which atopic individuals begin to display symptoms and airway reactivity. This observation has been extended by studies suggesting that the asthma-associated phenotypes in children with indoor allergen specific IgE are attenuated by IgG of the same specificity [39]. The mechanisms for these interactions in childhood or their roles in asthma in later life are not clear. However an underlying mechanism is likely to be age-dependent “maturation” of relevant elements of the host response to allergens, which probably includes changes in relative IgE and IgG titres and in antibody affinity, both of which are known to change progressively with continuing exposure.

Despite our wealth of knowledge on the risk factors associated with childhood asthma, our understanding of the life course of asthma is limited. Longitudinal birth cohort studies, such as the Tucson Respiratory study and the Raine Study, are uniquely poised to contribute to knowledge of the persistence of asthma throughout childhood and into the early adult years, as well as to further our understanding of the risk factors associated with the development of late-onset asthma in young adults.

#### Limited information about persistent *versus* late-onset asthma in young adults

Atopy in childhood, along with concomitant low lung function, airway hyper-responsiveness and being female have consistently been linked to the persistence of asthma from childhood to early adult life. It is likely that a range of immunological, physiological and environmental factors contribute to an individual’s risk for the development of asthma in young adults, in addition to development of chronic obstructive pulmonary disease in later life [40, 41]. While longitudinal studies of clinical asthmatics (such as the Melbourne Asthma study [42]) provide a wealth of information on factors associated with the persistence and long-term implications of asthma, they are unable to offer insights into the factors associated with onset of asthma after puberty. The factors associated with the onset of asthma post-adolescence are not well understood. In contrast, longitudinal, population-based birth cohorts, such as the Raine Study, are able to determine the impact of lung function, AHR, atopy, and environment not only on the persistence of asthma throughout childhood and into the adult years, but also on the development of asthma and chronic obstructive pulmonary disease in early and later adult life.

### Sleep disorders

#### The importance of sleep disorders

Clinically significant obstructive sleep apnoea (OSA) is a common condition occurring in 1−2 % of children [43–45] and 2−4 % of the middle-aged population [46–48]. It is caused by repetitive, upper airway obstruction due to collapse of upper airway structures during sleep. Obstructive respiratory events are accompanied by repetitive oxygen desaturation and blood pressure surges, and are usually terminated by brief awakenings (electroencephalographic arousals). OSA impairs daytime function and is associated with major reductions in quality of life [49, 50], increased risk of motor vehicle accidents [51] and cardiovascular disease; including hypertension, cardiac failure and stroke and an increase in all-cause mortality [52–54].

#### Early life predictors of sleep disorders in young adults

It is crucial to understand the early life, developmental and environmental influences predisposing to abnormal breathing during sleep as many individuals are asymptomatic, yet still at increased risk of metabolic and cardiovascular disease. Unfortunately, there is a paucity of data exploring early life influences on the development of OSA and on the trajectories of obesity that lead to OSA. It is highly likely that these influences commence in utero and are further influenced by early life environmental exposures.

In children, there are substantial, cross-sectional data on risk factors for OSA, including prematurity [55] and maternal smoking [43], but few longitudinal studies [55]. Although adenotonsillar hypertrophy has, historically, been the predominant OSA risk factor in children, recent data show that BMI represents a new, important risk factor, with OSA risk increased by a factor of 3.5 for each standard deviation increase in BMI z-score [56]. Notably, the increased risk appears to commence in the adolescent years (age ≥ 12 years). Research undertaken in other fields has shown there may be critical periods in childhood where growth patterns and weight gain have strong influences on adult health outcomes [57, 58]. Recognition of perinatal risks predisposing to the subsequent development of OSA may allow anticipatory screening to prevent the development of adverse consequences.

In a Swedish cohort of 4 year old children, parental reports of restless sleep and mouth breathing were strongly associated with habitual snoring [59]. Further, habitual snorers had cephalometric changes including a narrower maxilla [59]. Hence, there appear to be early markers of obstructive sleep disordered breathing and early anatomical changes which may predispose to OSA later in life. The Raine cohort has collected parental reports of sleeping behaviours in the first few years of life which will help address the relationship between infant sleep behaviours and subsequent development of OSA in adulthood.

#### Limited information about sleep disorders in young adults

**Fig. 1:**
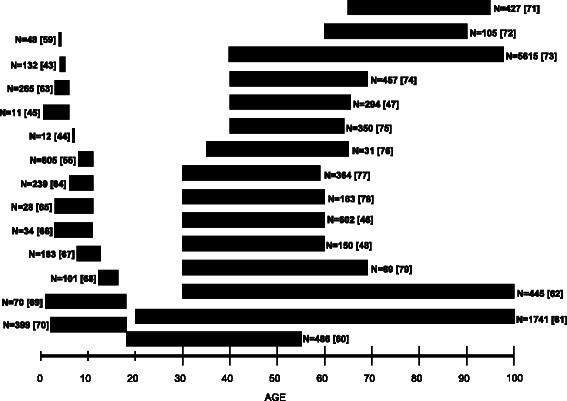
Obstructive sleep apnea prevalence studies, to date, showing the age range and sample sizes of study cohorts

The prevalence of OSA in young adults is unknown. This is due to a paucity of prevalence studies undertaken in this age group, as the focus to date has been almost exclusively on children [44, 45, 55] and cross-sectional studies among middle aged-adults [46–48, 52, 60], with few population-based studies with longitudinal follow-up of the natural history of OSA [52, 60]. The age ranges of most OSA prevalence studies, to date, are shown in Fig. 1 [43–48, 55, 59–79], which illustrates the focus to date on children and middle-aged and elderly adults. Despite using gold-standard methods (e.g. laboratory PSG in all or a subset of patients) studies have revealed considerable variability in OSA prevalence estimates for middle-aged men (1.2 % [61] to 19 % [62]) and women (1.2 % [80] to 15 % [62]). While these data have raised awareness of the high prevalence of undiagnosed disease [81] and the need for adequate consideration in terms of healthcare policy and research funding, the natural history of the disease remains poorly understood and disease onset and progression are largely unknown. Similarly, there are no data on the phenotype of OSA in young adults, which may differ from middle-aged individuals. Nor are there data on the early life risk factors for developing OSA in adulthood, including obesity.

### Physical activity and sedentary behaviour

#### The importance of physical activity and sedentary behaviour

Too little exercise (insufficient moderate/vigorous physical activity) is estimated to be the leading, modifiable cause of health burden in Australia, after tobacco [82]. Coupled with this, is the rapidly accumulating evidence that too much sitting (excess sedentary behaviour) is detrimentally associated with health, even in adults who undertake sufficient exercise for health benefits [83]. Taken together, these behaviours create an increasing burden of substantial public health concern [84].

Detailed, objective, international analyses have found that fewer than 10 % of adults achieve at least 30 min of moderate/vigorous physical activity accumulated in bouts of at least 10 min on at least 5 out of 7 days [85, 86]. This ‘inactivity’ has significant public health consequences. The World Health Organization has identified inactivity as the fourth largest global risk factor for mortality, causing an estimated 3.2 million deaths worldwide each year [87]. In Australia, inactivity has been estimated to cost the economy $14 billion annually [88].

There is now consistent and compelling evidence that time spent in sedentary behaviours as adults, independent of physical activity, is associated with all-cause mortality [89], cardiovascular disease [89], obesity [90], adverse metabolic profiles [91], and poor fitness in later life [92]. Importantly, associations remain even after adjusting for moderate/vigorous physical activity exposure [90, 93]. Recent evidence indicates that the adverse effects of sedentary behaviour are present in childhood [94], even in the early years [95], suggesting that understanding the life-course predictors of these behaviours is critical.

Furthermore, patterns of sedentary time accumulation are also important. Extended periods of uninterrupted sedentary time have been related to poor metabolic profiles in middle aged Australian adults (Australian Diabetes, Obesity and Lifestyle Study (AusDiab study) [93]), and to cardio-metabolic and inflammatory biomarkers in American (USA) adults (National Health and Nutrition Examination Survey (NHANES) cohort [96]). There is also evidence that light intensity activity, independent of moderate/vigorous physical activity, has important health benefits [97]. A recent study has also shown that light activity and sedentary time are highly negatively associated [97]. Thus, the whole spectrum of movement intensity, from sedentary through to vigorous, appears important to help prevent chronic disease and understand its development.

#### Limited information about sedentary behaviour in young adults

The sedentary behaviour habits of young adults (20–25 years) are largely unknown. This is due to a paucity of objective studies of sedentary behaviour undertaken in this age group, as the focus to date has been almost exclusively on children, middle-aged adults and elderly adults. No study has explicitly focused on the early adult years. The few studies with objective measures, that have included a proportion of young adults, have been quite broad in their age division categories: 20–30 years in the NHANES cohort from the USA [98]; 20–35 years in Belgium [99]; and 20–39 years in Canada [86]. Such age ranges limit the ability of these studies to inform influences on sedentary behaviour in young adults. Whilst the ongoing, longitudinal AusDiab study [93, 97] has included 25 year-olds at baseline, there were no objective measures obtained at this time point. Inactivity patterns vary with age and are influenced by different factors at different ages [100]. Therefore, there is a need for a focus on specific age ranges – in particular the critical period of young adulthood. Yet, thus far, there are no longitudinal studies utilizing life-course biological, psychological, social, behavioural and context variables to predict physical inactivity and sedentary behaviour in young adults. The Raine Study has a wealth of longitudinal early life predictors that may help explain the amounts and patterns of objectively measured physical activity and sedentary patterns among young adults.

#### Early life predictors of physical activity and sedentary behaviour in young adults

**Fig. 2:**
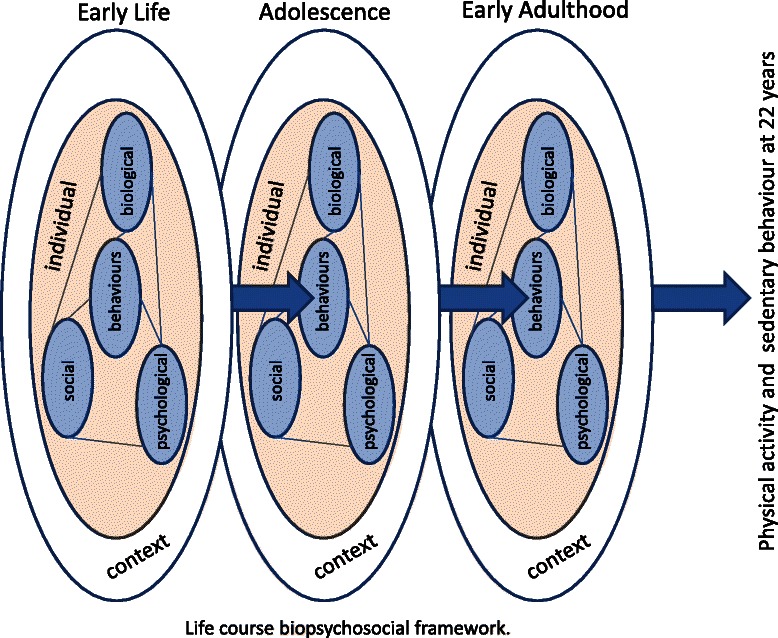
Life-course biopsychosocial framework

In order to develop interventions to promote physical activity and reduce sedentary behaviour in young adults, insight into determinants of these behaviours is necessary. However, much of the available evidence comes from cross-sectional studies. This has resulted in calls for high quality, prospective evidence [101]. Most early studies on health behaviour determinants have been informed by psychological theories of human behaviour with a strong focus on cognitive determinants [102]. More recent research has used a social-ecological framework [103] which acknowledges the importance of the environments in which people live. Contemporary models from ergonomics [104], occupational health [105], sedentary behaviour [101] and life-course epidemiological [106] fields also incorporate earlier sociotechnical systems and biopsychosocial models, thereby acknowledging the importance of individual characteristics and their interaction with other system elements which influence behaviours within a context over time. Adult research suggests many potential determinants of inactivity and sedentary behaviour including biological, physiological, and social factors. Determinants may be from occupation, transport, household and leisure contexts. However, there is limited similar research in young adults. Figure 2 represents a synthesis of these models as a framework for the Raine Study.

### Work productivity

#### The importance of young worker productivity

Productivity, along with increased supply of capital and labour, drives economic growth. The importance of productivity to the nation goes beyond purely economic terms [107] given it is a determinant of social welfare status [108], contributes to funding of societal institutes (e.g. law and order) [109], drives long-term prosperity [110] and ensures ongoing improvements in standard of living.

The aging population, both globally and in Australia, is placing and will continue to place greater burden on workers [111]. For example, it is projected that by 2056 there will be a 3:1 ratio of working adults for every older person, in contrast to the present ratio of 5:1 [112]. Young Australians (20–34 years) currently constitute the largest proportion of the civilian labour force (22 %) [113]. While Australian Government strategy has focused on increasing workforce participation in target groups (the aged, mothers, non-working males of working age) and through immigration [111], there will be growing pressure on younger workers to increase their productivity to support Australia’s standard of living. The importance of young workers will continue to increase into the future, as the (positive or negative) attributes of their productivity accumulates over a lifetime of workforce participation.

Health has been recognised as a key contributor to productivity [108]. Weil [114] estimated a worker in good health in a low mortality country would be about 70 % more productive than a worker suffering from ill health in a high mortality country. Additionally, major contributors to presenteeism were depression (21 %), allergies (17 %), hypertension (13 %), diabetes (12 %), spinal pain (7 %) and asthma (7 %) [115]. Bloom [116] concluded that ‘health is a vitally important form of human capital and deserves the same level of attention…as is currently paid to the accumulation of physical capital and education.” However, such considerations have been given limited attention.

Young workers are a key productivity resource now and into the future. Strategies to increase younger worker productivity have focused on education, training and transition into the workforce [117]. There is a common perception that health may not be a major problem for young workers, compared to older workers. However, young adults in Australia have a high prevalence of conditions such as spinal pain (15 %) [118], sleep problems (26 %) [119] and mental health disorders (26 %) [118] as well as poor health behaviours such as alcohol misuse (27−32 %%) [120]. These common health problems affecting young adults are all known negatively to impact on work productivity [121–123].

#### Limited information on young worker productivity

Absenteeism (absence of an individual from work) is the commonly used measure of the quantity of productive time lost. Presenteeism (reduction in productivity while an individual remains at work) is increasingly being recognised as a critical component in productivity loss as it deals with the quality of productive time. Since presenteeism is estimated to result in 3 to 7 times the productivity loss of absenteeism [124] and health conditions typically have a much greater impact on presenteeism than absenteeism [122, 125] it is essential that presenteeism be included in any modelling of health-related work productivity loss.

Given the importance of young worker productivity, a robust estimate of the magnitude of health related work productivity loss is critical. Current worker’s compensation data suggest musculoskeletal and mental health disorders are important conditions for young worker productivity loss as they account for 80 % of serious absenteeism claims (from 2008–09) [126]. However, no data on the full, work productivity loss for young workers across the range of common health conditions have been reported. Whilst several countries monitor national workers’ compensation absenteeism figures, there are concerns that those data present a biased estimate. Specifically, young workers underreport problems due to a range of reasons including potential negative impact on employment, lack of leave entitlements and lack of knowledge of rights and reporting systems [127, 128]. Australian Bureau of Statistics (ABS) data show only 38 % of injured workers under 25 years of age applied for compensation [127]. The Raine cohort provides an unbiased sample where data on health related productivity loss can be collected in a robust and prospective manner.

Knowing the size of the problem of health related work productivity loss in young workers, and which health conditions contribute most, is important. However, a clear understanding of what young workers with high and low productivity ‘look’ like is needed, to encourage high productivity and address low productivity. Modifiable health disorders such as back/neck pain [129], psychological distress [122], workplace injury and obesity [130] are all known to increase the risk of absenteeism and presenteeism. Health related lifestyle factors/behaviours such as sleep [123], physical activity [121] and drug use [122] also have a significant impact on work productivity. Importantly though, the prevalence of disorders [118] and health behaviours [131] varies with age. Despite this, health conditions and lifestyle factors profiles of high and low productivity young workers are not known.

#### Early life predictors of health related work productivity loss in young workers

Little is known about the relationship between early life health factors and young worker productivity. Childhood health appears to play a role in adult workforce participation [132] while childhood poverty is a predictor of reduced productivity later in life [133]. Further, spinal pain [134] and mental health [135] problems frequently develop in early life and track into adulthood. Thus, pre-adult factors may be important to young worker productivity. The Raine Study cohort provides a unique opportunity to examine these relationships, as it is not only representative of 22 year old adults, but also has an unparalleled richness and breadth of health related data spanning pre-birth to adulthood.

### Aims

The Raine Study 22 year follow-up had the following aims:

1. To describe the prevalence of critical health issues and behaviours in young adulthood;
2. To characterise the phenotypes of critical health issues and behaviours in young adulthood; and,
3. To evaluate the gestational, childhood and adolescent risk factors for critical health issues and behaviours in young adulthood.

## Methods

### Study design and sample

The study design was a prospective follow-up of an established pregnancy cohort at age 22 to 23 years. The study sample consisted of all active members of the cohort (*n* = 2,086 or 72.7 % of the original cohort of 2,868), that is those who had previously provided consent to be contacted for follow-up and were not deceased (*n* = 40), had not withdrawn (*n* = 566), or otherwise been lost to follow-up (*n* = 176).

### Ethics approvals

Approval for the 22 year follow-up was obtained from the Human Research Ethics Committees at the University of Western Australia (RA/4/1/5202) and Curtin University (HR67/2013). The study was conducted in accordance with the Declaration of Helsinki and informed consent was obtained from all participants.

### Measures

#### Spinal Pain

##### Pain questionnaire

Pain location, frequency, duration and intensity assessment was assessed with the Nordic Questionnaire which has demonstrated reliability and validity [136], with modifications based on subsequent research [137–139]. The impact of spinal pain on daily activity and physical activity, as well as on health care use, medications taken, health professionals visited [140] and work/study days lost [141] was also be assessed.

##### Back muscle endurance

Participants completed a prone trunk hold, the Biering-Sorenson test [142], which has high reliability in young people [143] and displays good validity [144].

##### Tissue sensitivity

Pain thresholds to both pressure and cold were collected using standard protocols [145]. Following sensation checks, pressure pain thresholds were tested 4 times on each location (right wrist, neck, leg and lumbar spine) using a Somedic digital algometer. Cold sensation threshold testing followed by cold pain threshold was tested 4 times on the dorsal aspect of the right wrist using a Somedic thermal stimulator.

#### Asthma

Previous and current respiratory symptoms and allergy information were assessed by validated, standardised questionnaires. Lung function was assessed by spirometry and the forced oscillation technique, and airway hyper-responsiveness to an inhaled mannitol challenge was also performed. Airway inflammation was assessed by exhaled nitric oxide and cellular studies were undertaken from induced sputum. Atopy to a range of common aeroallergens was assessed with skin prick tests complimented by measurement of total and specific IgE titres from serum samples. Participants were requested to withhold antihistamines and all asthma medications for 72 h prior to testing, and were questioned to verify medication withholding on the day of testing.

##### Respiratory questionnaire

Current, recent and previous respiratory and allergic symptoms and disease were assessed, using validated questionaries modified from the International Study of Asthma and Allergy in Children (ISAAC) [146] and based on those utilised by previous Raine Study follow-ups [32, 147] and the Busselton Healthy Ageing Study [35].

##### Spirometry

Spirometry was measured as per international guidelines [148] using a KOKO spirometer (inSpire Health Inc. USA) and FVC, FEV_1_, FEV1/FVC and FEF_25–75_ reported. Data were reported as both absolute and predicted values (expressed as z-scores) derived from the Global Lung Function for Spirometry reference ranges [149] the validity of which has been confirmed in a contemporary Australasian population [150].

##### Forced oscillation

The mechanical impedance of the respiratory system (Zrs) was measured with the modification of the FOT [151]. Briefly, a small-amplitude signal between 4 and 26 Hz was delivered to the subject through a mouthpiece *via* a low-deadspace wave tube. Averaged Zrs spectra in each subject were used to obtain total respiratory resistance and compliance. In addition to average values, the within-breath changes in Zrs, as functions of tidal volume and flow, during tidal breathing and during a slow inspiration to total lung capacity were also determined.

##### Airway hyper-responsiveness

The mannitol challenge (Pharmaxis Ltd, Frenchs Forest, NSW, Australia) test protocol consisted of inhalations of control (empty capsule) and increasing doses of mannitol to a maximal cumulative dose of 635 mg, per standard guidelines [152]. After each dose, subjects were instructed to expectorate any sputum present into a Petri dish. Inhalation of successive doses continued until the last dose was taken, or a positive response recorded. A positive response was defined as a 10 % fall in FEV_1_ between doses, or a 15 % fall from baseline FEV_1_ (PD15). Responsiveness was defined using the PD15 and the dose response slope.

##### Exhaled nitric oxide

Exhaled nitric oxide was measured as a surrogate for airway inflammation. Briefly, subjects were seated and asked to inhale NO-free air to total lung capacity and immediately to exhale through a mouthpiece, according to international guidelines [153]. Flow was maintained at 50 mL/s using a feedback system displayed by the equipment software (Ecomedics, Switzerland). A minimum of 3 acceptable measurements that varied by less than 10 % were obtained.

##### Induced sputum

Sputum samples were obtained during the mannitol challenge test, as described above and reported previously [154, 155]. Within 2 h of collection the pooled sputum sample was dispersed with Sputolysin (Calbiochem) and total and differential cell counts made from stained cytospin slides, per international guidelines [156, 157]. CD45+ cells were selected using Dynabeads (Dynal), resuspended in RNALater (Ambion) for preservation of nucleic acids and stored at −20 °C for future studies. Any remaining sputum fraction was cryobanked for future metagenomic studies to examine bacterial colonisation of the airways.

##### Blood collection

A 100 ml sample of peripheral blood was collected into a tube containing preservative-free heparin and processed within 2 h. Plasma was isolated and aliquots stored at −80 °C for measurement of antibodies and for future studies. Following removal of plasma, peripheral blood mononuclear cells were isolated within 18 h of this step using Lymhoprep. After counting, peripheral blood mononuclear cells were suspended in medium to 16×10^6^ cells/ml, and 0.5 ml aliquots prepared for cryopreservation and storage in a monitored gas phase liquid nitrogen storage tank. A single aliquot of whole blood was processed for a full blood count, including circulating eosinophils, neutrophils, macrophages, basophils and lymphocytes.

##### Skin prick testing

Skin prick testing was performed according to local guidelines [158]. Allergens used were: Cat fur and pelt, dog hair, dust mite-*Dermatophagoides farinae*, dust mite-*Dermatophagoides pteronyssinus*, perennial rye grass, grass mix, American cockroach, *Alternaria Tenuis, Aspergillis Fumigatus*, whole cow’s milk, egg white, Histamine Hydrochloride 10 mg/ml positive control, and glycerine/saline negative control. The length and widths of each resulting wheal for each allergen and the controls was recorded. A positive result indicating sensitisation was defined for each wheal that exceeded the negative control by >3 mm [32].

#### Physical activity and sedentary behaviour

##### Accelerometers

Tri-axial Actigraph GT3X+ monitors (Florida, USA) were used to capture date and time-stamped activity data. These portable lightweight (19 g; 4.6× 3.3× 1.5 cm) water resistant accelerometers collected movement data in raw format, allowing a range of sophisticated analytic options. The GT3X+ is currently being used in other major studies, thus use of this device enabled direct comparison with other datasets.

Data collection and analysis protocols follow best practice guidelines [159]. Participants were asked to wear two GT3X+ accelerometers continuously (24 h per day) on the right hip and non-dominant wrist for eight consecutive days, with the raw data collected at 30Hz. Sleep times, work times, and any removal times were recorded in a diary.

Initially, data from the vertical axis (equivalent to the previous GT1M Actigraph model –[160]), exported as 60 s epoch files, were used to derive key sedentary and inactivity measures across the whole day and during specific periods, such as work, and on weekends and weekdays. Time spent being sedentary (<100 counts per minute, cpm), in light-intensity activity (100-1951 cpm), and in moderate-to-vigorous intensity activity (>1951 cpm: MVPA) was considered. Similar, triaxial assessments will also be generated in the future [160]. Non-adherence to physical activity recommendations (achievement of 30 min of MVPA on at least 5 out of 7 days accumulated in bouts of 10 min or more [85] and the proportion of wake time spent in sedentary activity were determined. Patterns of sedentary time and MVPA were assessed with examination of bouts, including 10-min bouts of MVPA and prolonged bouts (>20mins; >30mins) of sedentary time, as well as the number of breaks in sedentary time (the number of transitions between a sedentary (<100 cpm) and active (≥100 cpm) time) [91].

##### Activity questionnaire

Time spent in physical activity and sitting, and in specific sedentary behaviours (e.g. computer use, television time) was assessed using the short form of the International Physical Activity Questionnaire [72] and Raine Study standard questions based on the Kaiser Family Foundation USA national survey [161] and the Young People’s Activity Questionnaire [162].

#### Health related work productivity

##### Work productivity loss

At 23 years of age was prospectively captured using the World Health Organisation’s recommended Health and Productivity Questionnaire (HPQ) [163]. The HPQ provides separate estimates of both absenteeism and presenteeism over the previous 4 week period for any health reason. Given inconsistencies in organisational data, self-report of absenteeism and presenteeism is the preferred method to enable consistent estimates across a community cohort and account for multiple, part-time and casual employment: an important consideration for younger workers. The HPQ has also demonstrated good one week test-retest reliability (*r* = 0.89) [164]. The scale has been used in around 30 countries and has an international master database of benchmark data. Absenteeism is presented as hours lost per month for each worker, from which an annualised estimate can be made. Validation studies show HPQ self-reports of absenteeism have good concordance with payroll records (r ~0.7) [164]. A small but consistent bias towards under-estimation of absence data by self-report is accounted for using a correction factor. Presenteeism was calculated for each worker in terms of hours lost per month, and as an annualised estimate, by combining hours worked with job performance rating. Validation studies show that HPQ self-reports of presenteeism correlate well with independent assessments such as work audits, supervisor ratings and peer ratings across a broad range of industries and occupations [164]. The common metric allows not only a consolidated total work productivity loss measure, but also facilitates cost modelling [165] based on salary conversion methods using a human capital approach, which expresses productivity loss as the product of lost work time and salary [166]. The questionnaire was completed at the time of the overnight sleep study and then online following a text message screening question prompt every 3 months for the following 12 months.

The HPQ also includes self-reported status for 28 health conditions covering 9 broad domains [122]. This study used a similar, self-reported list of health conditions to be consistent with those used in previous Raine follow-ups [167], which has good overlap with the HPQ for common conditions but also includes conditions potentially important for work productivity loss in this age-group but not covered by the HPQ, such as menstrual disorders. Self-report of conditions on such checklists has a high concordance with medical records [168].

#### Sleep

##### Polysomnography

Standard, overnight monitored polysomnography was performed in home-like bedrooms at the University of Western Australia’s Centre for Sleep Science. Measurements included electroencephalogram, electrooculograms, submental electromyogram, oronasal airflow, chest wall motion, and arterial oxygen saturation. All studies were monitored by a Sleep Scientist. Scoring for sleep stages, respiratory events and arousals were performed according to current American Academy of Sleep Medicine criteria [169].

##### Sleep questionnaires

The following sleep-related questionnaires were also administered: Pittsburgh Sleep Quality Index: a self-administered questionnaire that measures sleep quality and sleep disturbance retrospectively over a one month period; the Berlin questionnaire: a self-administered questionnaire that identifies individuals at risk for the sleep apnea syndrome; the Epworth Sleepiness questionnaire: a self-administered questionnaire regarding the levels of daytime sleepiness in various situations; and, the short Functional Outcomes of Sleep Questionnaire: a self-administered questionnaire designed to assess the impact of excessive sleepiness on multiple activities of everyday living (activity, vigilance, relationships, productivity, social outcome).

##### Sleep accelerometry

Wrist and hip accelerometry was undertaken for one week (see physical activity section for details).

##### Sleep diary

Sleep diary was given to each participant at the overnight sleep study in order to document sleep quality and sleep hours for a week in their normal lives (see physical activity section for details).

#### Additional variables

Additional variables were collected by questionnaire and physical assessment to continue collation of longitudinal measures regularly collected from the cohort, and other variables of particular interest to investigators. These are summarised as follows:

Sociodemographic variables collected by questionnaire included family structure, residential situation, education, occupation, income, and ancestry. Health variables collected by questionnaire included diagnosed disorders, medical history, medication use, and health services utilised. Lifestyle variables collected by questionnaire included sunlight exposure, diet, smoking, alcohol and other drug use. Quality of life was assessed using the SF-12. [170] Psychological variables included depression, anxiety and stress, assessed by the Depression, Anxiety and Stress Scale-short form (DASS-21) [171].

Physical assessments using established, standard methods included height (stadiometer, unshod), weight (electronic scale, light clothing), chest, waist and hip circumference (flexible tape), subscapular, triceps, supraspinale/suprailiac and abdominal skinfolds (Holtain calipers), blood pressure and resting heart rate (Dinamap). Additional anthropometric measures related to sleep issues included neck circumference, observations for Mallampati score and pharyngeal grade, and intra-oral and three dimensional facial photographs. Mole counts on one arm and skin wrinkling impression on dorsum of hand were also taken. Evening and morning urine samples were taken for later processing. Cognitive function was objectively assessed using subtests of the CogState (CogState Ltd, Melbourne, Australia), a computerized test battery developed in Australia, measuring speed of processing, sustained attention (vigilance), working memory and executive function, and and visual learning and recall. For each subtest, hit rates and mean reaction times provide sensitive indices of performance. The assessment is suitable for participants aged 6 to 106, and has been used to assess the cognitive consequences of a number of disorders [172].

### Procedures

The procedures followed standard practices used with this cohort since inception. At around the time of their 22nd birthday (2012–2014) each participant was contacted by telephone and had the details of this follow-up explained. Those wishing to participate were mailed questionnaires, participant information and a consent form prior to their scheduled appointment for a physical assessment session and overnight sleep study. The questionnaire took approximately 2 h to complete. The physical assessments took approximately 3 h to complete plus an additional one and a half hours for sleep study set up.

All data were entered into the Raine Study database and quality controlled using standard procedures. These new data were added to the existing database of over 85,000 phenotypic variables, 31 million genetic variables, and the 170,000 biological samples curated (Table 1).

**Table 1:** Longitudinal data available on Raine Study participants

	Age (years)											
variable	18 weeks	birth	1	2	3	5	8	10	14	17	20	22
parental	+	+	+	+	+	+	+	+	+	+		
genetic									+	+		
physical	+	+	+	+	+	+	+	+	+	+	+	+
mental		+	+	+	+	+	+	+	+	+	+	+
behavioural			+	+	+	+	+	+	+	+	+	+
environmental	+	+	+	+	+	+	+	+	+	+	+	+

### Analysis

#### Aim 1

Descriptive statistics with precision estimates will be used to report the prevalence of each critical health issue and behaviour using cross-sectional 22 year data.

#### Aim 2

Phenotypes, detailed profiles and patterns of each health issue, at 22 year will be described. Comparisons between groups with and without each health issue (e.g. atopic vs non-atopic asthma, active vs inactive) will be conducted using general linear models.

#### Aim 3

Early life predictors will be assessed using variables that have been measured repeatedly over the prior 20 years of the cohort to establish developmental trajectories for each phenotype. Specific risk factor variables were selected based on previous literature, exploratory correlations, and exploratory factor analysis. A generalised linear model framework (multivariable logistic regression) will explore the strength of association of these factors with the presence of each phenotype both univariately and collectively. Where repeated measures of a predictor are available (e.g. Body Mass Index), latent growth curve analysis will be used to characterise risk factor trajectory data for use in the logistic regression model [14]. For binary outcomes, logistic regression, with population-averaged approaches or random effects, will be used [173].

More detailed secondary analyses will be conducted specific to each health issue and non-parametric models used when necessary.

## Discussion

The 22 year follow-up of the Raine Study cohort provides unprecedented data on the prevalence, clinical picture (phenotype), risk factors and inter-relationships for multiple health conditions and behaviours among young adults.

### Prevalence

Disease prevalence data are vital to enable adequate planning by health authorities and to facilitate appropriate allocation of healthcare resources. The focus on young adults is also appropriate given the lack of data in this age-group across the health conditions, and the potential positive impact of early identification on public health and clinical measures to improve quality of life and alleviate risk for long-term health outcomes. For example, international evidence shows that young adulthood is a critical time for development of lifelong behaviours but also for increasing inactivity and sedentary behaviour. Therefore, there is considerable potential for the findings from this study favourably to impact on long-term musculoskeletal, respiratory, cardiovascular, metabolic and other health outcomes by informing public health planning and the development of early preventative strategies.

### Phenotype

The study had adequate power to define the clinical features and phenotype of health conditions, behaviours and consequences in young adults. Such information can be used to educate primary care physicians in early case identification. Detailed measurement at 22 years of age also provides an excellent baseline for subsequent research not only to track these behaviours into middle age and beyond, but also to test hypotheses about the contribution that early symptoms and behaviours make to the complex pathways to many chronic diseases in adulthood.

### Risk factors

The study can identify unique and cross-cutting risk factors for multiple health conditions. For example, obesity at an early age may contribute to OSA, asthma, increased spinal pain and increased sedentary time. The Raine cohort has prospectively collected detailed longitudinal data and provides an ideal platform to evaluate the role played by developmental and environmental factors. Findings from this study will have important public health planning implications, potentially leading to the development of early preventative strategies including early identification of high-risk individuals and, ultimately, targeted treatment, which may prevent disease onset or modify disease course. Young adults are also frequently becoming parents, thus assisting improvements in their health and related behaviours is likely to have benefits not only for their own health but also for the health of their offspring.

### Inter-relationships

These five areas of focus for the 22 year follow-up are likely to be related. For example, physical activity and reduced sedentary time are associated with spinal pain [174], asthma [175] and sleep [176]. Further, sleep disorders such as obstructive sleep apnea (OSA) are likely to be influenced by physical activity. In middle aged adults there is an association between the severity of OSA and reported hours exercised per week, independent of traditional covariates, including BMI [177], suggesting that physical activity may be an additional, potentially modifiable, risk factor. These relationships may also be bidirectional. For example, while high levels of physical activity may lead to back pain, disabling LBP is also associated with activity avoidance and physical deconditioning [178]. In addition to direct relationships, there are likely to be several common, early life and young adulthood risk factors. For example, obesity is a risk factor that may influence pain, asthma, sleep disorders and limit physical activity and work productivity *via* a number of different mechanisms [179]. All of these health conditions and behaviours may contribute to work productivity.

### Conclusion

A major focus of health and medical research today is on developing a better understanding of the underlying mechanisms, and addressing the rising prevalence, of lifestyle-related chronic diseases whilst encouraging healthy lifestyles. The Raine Study is uniquely placed to contribute to this *via* its breadth and depth of multidisciplinary information. There are few community cohorts that have been followed from before birth into adulthood in such rich detail. This continuing examination of the Raine cohort in early adulthood and beyond will provide critical information on the development of multiple, chronic health conditions and their impact on society.

## Abbreviations

BMI: Body Mass Index
FEF: forced expiratory flow
FEV: forced expiratory volume
FVC: forced vital capacity
HPQ: Health and Productivity Questionnaire
OSA: Obstructive Sleep Apnea
PSG: polysomnography
Zrs: mechanical impedance of the respiratory system.
